# Direct observation of accelerating hydrogen spillover via surface-lattice-confinement effect

**DOI:** 10.1038/s41467-023-36044-8

**Published:** 2023-02-04

**Authors:** Yijing Liu, Rankun Zhang, Le Lin, Yichao Wang, Changping Liu, Rentao Mu, Qiang Fu

**Affiliations:** 1 State Key Laboratory of Catalysis, Dalian Institute of Chemical Physics, Chinese Academy of Sciences, 116023 Dalian, China; 2 University of Chinese Academy of Sciences, 100039 Beijing, China; 3 Zhang Dayu School of Chemistry, Dalian University of Technology, 116024 Dalian, China; 4 CAS Key Laboratory of Science and Technology on Applied Catalysis, Dalian Institute of Chemical Physics, Chinese Academy of Sciences, 116023 Dalian, China

**Keywords:** Catalytic mechanisms, Heterogeneous catalysis, Scanning probe microscopy

## Abstract

Uncovering how hydrogen transfers and what factors control hydrogen conductivity on solid surface is essential for enhancing catalytic performance of H-involving reactions, which is however hampered due to the structural complexity of powder catalysts, in particular, for oxide catalysts. Here, we construct stripe-like MnO(001) and grid-like Mn_3_O_4_(001) monolayers on Pt(111) substrate and investigate hydrogen spillover atop. Atomic-scale visualization demonstrates that hydrogen species from Pt diffuse unidirectionally along the stripes on MnO(001), whereas it exhibits an isotropic pathway on Mn_3_O_4_(001). Dynamic surface imaging in H_2_ atmosphere reveals that hydrogen diffuses 4 times more rapidly on MnO than the case on Mn_3_O_4_, which is promoted by one-dimension surface-lattice-confinement effect. Theoretical calculations indicate that a uniform and medium O-O distance favors hydrogen diffusion while low-coordinate surface O atom inhibits it. Our work illustrates the surface-lattice-confinement effect of oxide catalysts on hydrogen spillover and provides a promising route to improve the hydrogen spillover efficiency.

## Abstract (web summary)

Hydrogen spillover is a surface phenomenon encountered in catalytic reactions. Here the authors show that the surface-lattice-confinement effect can regulate the hydrogen spillover directions and accelerate spillover rates. It is intrinsically related to the local surface geometries and coordination numbers of surface O sites.

## Introduction

The “hydrogen spillover”, first evidenced in experiments by Khoobiar in 1964^1^, depicts the dynamic migration of surface adsorbed hydrogen species from hydrogen-rich sites to hydrogen-poor sites. Considering its great potential in H-involving reaction processes, including methanol synthesis^2^, Fischer-Tropsch synthesis^3^, hydrogenations^4,5^, hydrogen storage^6^, etc., hydrogen spillover comes to a hot research topic among scientists, who intend not only to interpret it but also to exploit it for improving reaction performance and functionalizing materials^7,8^. In many cases, the migration of spilt hydrogen atoms is determined to be the rate-determining step in hydrogenation reactions. For example, the higher diffusion rates of the hydrogen species on chromium oxide than that on zinc or aluminum oxides corresponds to the following hydrogenation rates order: Cr_2_O_3_ ≫ ZnO ≈ Al_2_O_3_^9^. For spatially separated Pt and Co nanoparticles on SiO_2_, the hydrogen atoms can diffuse across the SiO_2_ support to reduce surface oxygen-containing species on Co nanoparticles, and thus promoting CO_2_ methanation reaction^10^. Tan et al. also found that an enhanced hydrogen spillover from Pt to Fe over the SiO_2_ support with the assistance of gaseous oxygenate molecules containing carbonyl functional group significantly accelerates the rate of hydrodeoxygenation of pyrolysis bio-oil vapor^11^.

Although hydrogen spillover has been implicated in a variety of scientific and technological fields, it has proven to be challenging to demonstrate the dynamics and kinetics of spilt hydrogen species. The blossoming of in situ characterization technologies has pushed forward the monitoring of hydrogen spillover^8^. By using X-ray photoelectron spectroscopy (XPS), the changes of surface oxidation states during hydrogen spillover on oxides have been investigated^12^. However, tracking hydrogen spillover at nanoscale is highly demanding in order to reveal the active sites. Employing nanometer-scale resolved X-ray absorption spectroscopy in a X-ray photoemission electron microscope, Karim et al.^13^ concluded that hydrogen spillover on Al_2_O_3_ is slower and limited to shorter distance than that on reducible TiO_2_. Tip-enhanced Raman spectroscopy combined with scanning tunneling microscopy (STM) has been employed to study hydrogen spillover at nanoscale^14,15^. Taking advantage of low-temperature STM, the diffusion of hydrogen atoms from surface Pd sites to surrounding Cu(111) and Ag(111) surface domains on bimetallic catalysts has been directly observed^16,17^. However, microscopic understanding of the intrinsic properties of oxide surfaces that determine the hydrogen spillover process is still missing, thus calling for in situ/operando surface characterizations in H_2_ atmosphere and at atomic scale.

Manganese oxides (MnO_*x*_) have been widely utilized in H-involving reaction processes, such as hydrogenation^18^ and hydrogen storage^19^, during which MnO_*x*_ with different structures usually exhibit divergent performance.

Here, we constructed two well-defined MnO_*x*_ surfaces, stripe-like MnO(001) and grid-like Mn_3_O_4_(001) monolayers on Pt(111) substrate, whose surface structures differ immensely from each other. High pressure STM (HP-STM) and XPS experiments reveal that the spillover direction is effectively regulated by the surface structure and the rate is largely promoted by the one-dimension (1D) surface-lattice-confinement effect. Density functional theory (DFT) calculations demonstrate that the differences of hydrogen diffusion barrier are essentially related to the local surface geometries and coordination numbers of surface O sites in the Mn oxide monolayers.

## Results

### Construction of monolayer MnO(001) and Mn_3_O_4_(001)

**Fig. 1:**
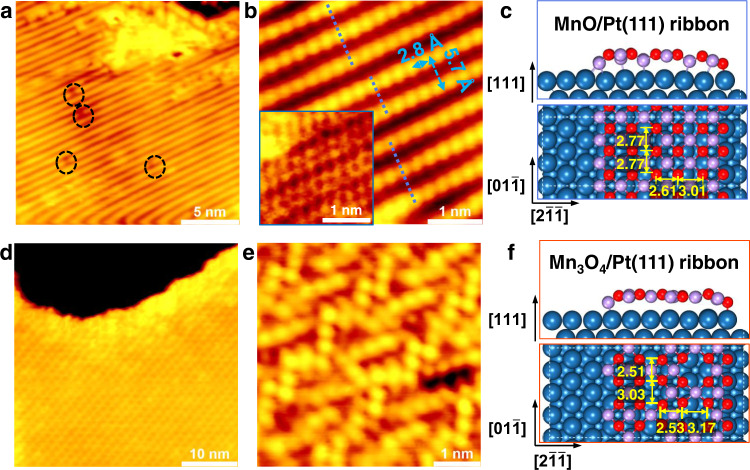
STM images and proposed models of manganese oxides on Pt(111). **a** STM image of as-prepared stripe-like MnO(001)/Pt(111) surface. **b** Atomic corrugation of stripe-like MnO(001) and the inset in (**b**) shows the atomic-resolution STM image of the surface. **c** A (6 × 3) MnO ribbon supported on the (5√3 × 3) Pt(111) substrate, which is equivalent to a moiety from the reported (19 × 1) reconstruction. The orthogonal MnO monolayer can be truncated along the rock-salt MnO(001) surface. **d** STM image of as-prepared grid-like Mn_3_O_4_(001)/Pt(111) surface. **e** Atomic-resolution STM image of the grid-like Mn_3_O_4_(001). **f** An Mn_3_O_4_ ribbon supported on the (5√3 × 4) Pt(111) substrate. The Mn_3_O_4_ monolayer is derived from a reconstruction of the spinel Mn_3_O_4_(001) truncation. Scanning parameters: (**a**, **b**) *I*_*t*_ = 0.090 nA, *V*_*s*_ = 0.969 V; inset in (**b**) *I*_*t*_ = 0.280 nA, *V*_*s*_ = 0.009 V; (**d**) *I*_*t*_ = 0.100 nA, *V*_*s*_ = 1.400 V; (**e**) *I*_*t*_ = 0.240 nA, *V*_*s*_ = 0.008 V. O: red; Mn: light violet; Pt: dark blue. Note that the distances denoted by yellow numbers are obtained by correcting the calculated values (2.82 Å) to align to the experimental Pt lattice constant (2.77 Å).

MnO_*x*_ nanoislands were prepared by reactive evaporation of Mn in O_2_ atmosphere on Pt(111) substrate. Depositing Mn atoms in 1 × 10^−7^ mbar O_2_ at 423 K followed by annealing in vacuum to 700 K produces 0.6 monolayer (ML) MnO_*x*_ islands with ~1.8 Å apparent height. The island surface exhibits a characteristic uniaxial stripe structure with an averaged distance of ~5.7 Å ($[2\bar{11}]$ direction) and a corrugation of 2.8 Å in each stripe ($[01\bar{1}]$ direction). As indicated by black dashed circles in Fig. 1a, a small number of dark spots, which might be oxygen vacancies, are distributed randomly on the island surface. Closer inspection of the atomic periodicity indicates that the stripes are in groups of 3 or 2 (a 3-2-3 sequence is shown in Fig. 1b), between which a shift close to half a unit cell exists along $[01\bar{1}]$ direction. The atomic STM image inserted in Fig. 1b indicates that each stripe consists of two Mn or O rows, which is consistent with the previously reported structure of MnO(001)/Pt(111) surface^20^. Accordingly, the atomic model structure is proposed in Fig. 1c. While depositing Mn atoms in 5 × 10^−7^ mbar O_2_ at 373 K followed by annealing in vacuum to 600 K, a grid-like surface structure (Fig. 1d) with ~2.0 Å apparent height and 0.7 ML coverage can be obtained. The atomic structure (Fig. 1e) is almost the same as that of Mn_3_O_4_/Au(111) surface prepared in “oxygen rich” regime^21^, where the bright features are arranged in a zigzag-like pattern with a spacing of ∼3.0 Å.

**Fig. 2:**
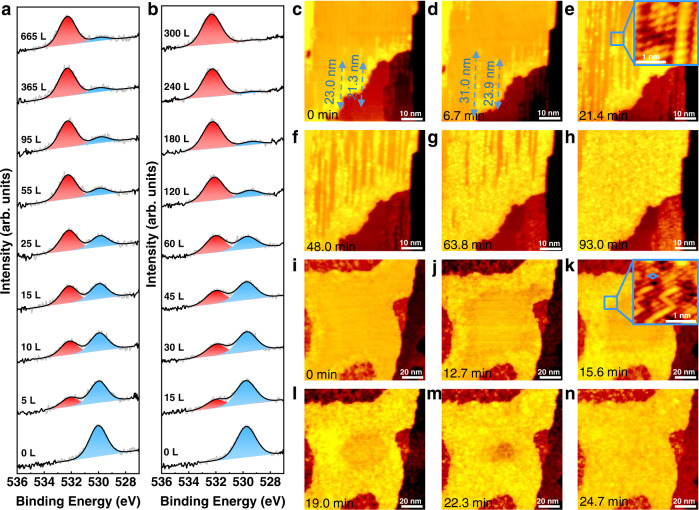
The spillover processes on stripe-like MnO/Pt(111) and grid-like Mn_3_O_4_/Pt(111) surfaces. **a**, **b** XPS O 1*s* spectra of stripe-like MnO and grid-like Mn_3_O_4_ surfaces exposed to different amount of D_2_ at room temperature. A time-lapse sequence of STM images of (**c–h**) stripe-like MnO and (**i–n**) grid-like Mn_3_O_4_ in H_2_ atmosphere at room temperature. Insets in (**e**) and (**k**) show atomic-resolution STM image of partially hydroxylated stripe-like MnO surface and partially hydroxylated grid-like Mn_3_O_4_ surface, respectively. The partial pressure of H_2_ atmosphere from (**d**) to (**h**) is ~2 × 10^−8^ mbar, and from (**j**) to (**n**) is 2.5 × 10^−7^ mbar. The exposure time is stamped in the lower left corner. Scanning parameters: (**c**, **d**) *I*_*t*_ = 0.090 nA, *V*_*s*_ = 0.831 V; (**e–h**) *I*_*t*_ = 0.090 nA, *V*_*s*_ = 0.937 V; inset in (**e**) *I*_*t*_ = 0.360 nA, *V*_*s*_ = 0.008 V; (**i**) *I*_*t*_ = 0.100 nA, *V*_*s*_ = 1.030 V; (**j–n**) *I*_*t*_ = 0.110 nA, *V*_*s*_ = 1.030 V; inset in (**k**) *I*_*t*_ = 0.100 nA, *V*_*s*_ = 0.019 V.

The bottom curves in Fig. 2a, b present XPS O 1*s* spectra of as-prepared stripe-like and grid-like MnO_*x*_/Pt(111) surfaces, respectively. O 1*s* peak of the as-prepared stripe-like MnO_*x*_ (Fig. 2a) is located at 529.9 eV, which is consistent with the peak position of lattice O (O_L_) of MnO^22^. Then, the XPS O 1*s*/Mn 2*p* signal ratio from the MnO(001)/Pt(111) surface was normalized to 1.00. O 1*s* peak of the as-prepared grid-like MnO_*x*_ (Fig. 2b) is located at 529.7 eV, 0.2 eV lower than that of MnO, which can be assigned to O_L_ of Mn_3_O_4_^22^. The XPS O 1*s*/Mn 2*p* signal ratio of the grid-like MnO_*x*_ is determined to be 1.31. Accordingly, the grid-like surface can be assigned to distorted Mn_3_O_4_(001) on Pt(111). Its model is proposed in Fig. 1f.

### Hydrogen spillover on stripe-like MnO(001) and grid-like Mn_3_O_4_(001) in H_2_

XPS O 1*s* spectra of stripe-like MnO surface exposed to increasing amount of D_2_ are shown in Fig. 2a. After the sample is exposed to 5 L D_2_, a new peak located at 532.1 eV emerges, which can be assigned to OD^23^. The peak area of O_L_ decreases by 86% while that of OD increases when the D_2_ exposure amount increases from 0 to 365 L, indicating the transformation of O_L_ to OD. When continuing to increase the D_2_ dosing to 665 L, the peak areas of OD and O_L_ remain almost unchanged, with their peak positions located at 532.3 and 529.6 eV, respectively. The maximum hydroxylation degree is calculated to be 88%, implying that MnOD_*x*_ is formed after the exposure of D_2_ to MnO^24^.

HP-STM was employed to investigate the hydroxylation process in situ. For the as-prepared stripe-like MnO surface, a few bright lines with ~1.1 Å apparent height exist on the surface as kept in UHV (Fig. 2c), which might be induced by H_2_ in the background. The hydroxylation process was further investigated under 2.0 × 10^−8^ mbar H_2_. Most of these bright lines start from the edge of the MnO island and extend to the middle following the shorter cell vector $[01\bar{1}]$ direction. The bright lines continue to grow along 1D pathway with the increasing H_2_ dosage (Fig. 2c–h, for the full series see Supplementary Movie 1). As indicated by the blue dashed lines from Fig. 2c, d, the length changes of each bright line vary, indicating that the spillover rates among all lines are different, which might result from the drag of oxygen vacancies on the hydrogen diffusion. Inset in Fig. 2e implies that the hydroxylation of stripe-like MnO surface is accompanied by surface reconstruction from the tetragonal to hexagonal symmetry.

In order to investigate the origin of hydroxylation, a 1.2 ML stripe-like MnO overlayer was grown for comparison (Supplementary Fig. 1a). Then, the sample was exposed to 1 × 10^−6^ mbar H_2_ and no bright lines appeared on the surface (Supplementary Fig. 1b), indicating that hydroxylation cannot take place without bare Pt surface. Therefore, it can be inferred that H_2_ dissociates on bare Pt region of the submonolayer MnO/Pt(111) surfaces and then dissociative hydrogen atoms spillover from Pt to MnO islands^25^.

XPS O 1*s* spectra of grid-like Mn_3_O_4_ exposed to D_2_ are shown in Fig. 2b. Upon the exposure of 15 L D_2_, a new peak appears at 531.7 eV, which can be assigned to OD^23^. As the D_2_ exposure increases from 0 to 240 L, the peak area of O_L_ decreases by 93% while that of OD increases. When D_2_ exposure further increases to 300 L, XPS O 1*s* peak areas of O_L_ and OD remain almost unchanged, with their peaks located at 529.4 and 532.4 eV, respectively. Coincidently, the peak positions are consistent with those from the hydroxylated stripe-like MnO surface (Fig. 2a). The maximum degree of hydroxylation is determined to be 96%, indicating that MnOD_*x*_ should be formed. As shown in Supplementary Fig. 2, the ratio of total O to Mn signal is calculated to be 1.13, decreasing by ~13% compared with that of the as-prepared Mn_3_O_4_. This change of the O/Mn ratio suggests that the hydroxylation of grid-like Mn_3_O_4_ surface is accompanied by H_2_O generation^26,27^. Notably, we find that H_2_O begins to be generated as the hydroxylation degree of Mn_3_O_4_ is increased to ~50%.

The hydroxylation process of grid-like Mn_3_O_4_ surface was also investigated by HP-STM in H_2_ at room temperature. The apparent height of the rim of grid-like Mn_3_O_4_ island is ~0.6 Å higher than the center area when the sample is exposed to H_2_ (Fig. 2i). Inset in Fig. 2k shows that the brighter rim is hexagonally symmetric (highlighted by a blue rhombus) with ~3.3 Å atomic distance. When H_2_ partial pressure was increased to 2.5 × 10^−7^ mbar, the brighter area continues to extend into the Mn_3_O_4_ island center with the hydroxylation front almost parallel to the edge of the island (Fig. 2i–n, for a time series see Supplementary Movie 2), which differs from the 1D spillover pathway on the stripe-like MnO island (Fig. 2c–h). In addition, the hydroxylation cannot take place on the 1.2 ML grid-like Mn_3_O_4_/Pt(111) surface (Supplementary Fig. 1c, d), indicating that the hydroxylation is also caused by hydrogen spillover from bare Pt(111) surface region to Mn_3_O_4_ islands.

**Fig. 3:**
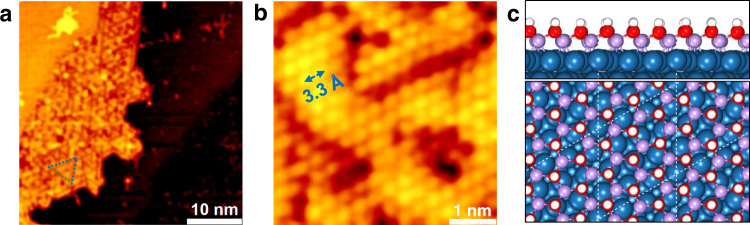
Structure of the hydroxylated Mn oxide surface. **a** STM image of a hydroxylated surface. **b** Atomic-resolution STM image of the hydroxylated surface. Scanning parameters: (**a**) *I*_*t*_ = 0.100 nA, *V*_*s*_ = 0.999 V; (**b**) *I*_*t*_ = 0.210 nA, *V*_*s*_ = 0.004 V. **c** Proposed MnOH_*x*_/Pt(111) surface structure. H: white; O: red; Mn: light violet; Pt: dark blue. The white dashed line indicates the boundary of the unit cell.

STM images and LEED patterns of hydroxylated MnO and Mn_3_O_4_ surfaces are shown in Fig. 3 and Supplementary Fig. 3. Highlighted by blue dashed lines (Fig. 3a), the island surface presents hexagonal symmetry with regular triangles. The atomic distance is ~3.3 Å, exhibiting 19% lattice mismatch between the MnOH_*x*_ overlayer and Pt(111) substrate. Since hydroxylation degree of MnO and Mn_3_O_4_ can both reach ~90%, the bright features in Fig. 3b can be assigned to OH species and the atomic model of MnOH_*x*_/Pt(111) surface is thereby proposed in Fig. 3c. It should be noted that the hydroxylation of manganese oxide monolayers is not reversible. Zhang et al.^28^ have shown that the desorption product of MnOH_*x*_ film is H_2_O instead of H_2_.

### Kinetics of hydrogen spillover on MnO(001) and Mn_3_O_4_(001) surfaces

**Fig. 4:**
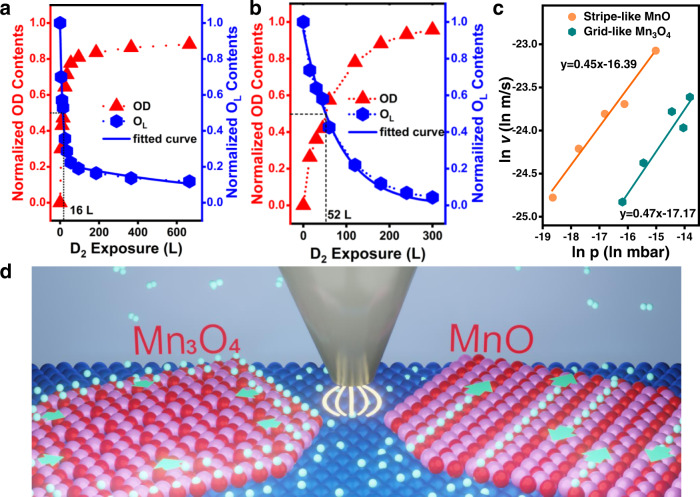
Hydrogen spillover rates on MnO and Mn_3_O_4_. OD and O_L_ contents derived from the XPS O 1*s* areas of (**a**) stripe-like MnO and (**b**) grid-like Mn_3_O_4_ surfaces with different amount of D_2_ exposure. **c** Dependence of spillover rates of stripe-like MnO and grid-like Mn_3_O_4_ surfaces on H_2_ partial pressure. The logarithms of spillover rates *vs*. logarithms of ${p}_{{H}_{2}}$. **d** Schematic of HP-STM using a STM tip to probe the hydrogen spillover on MnO and Mn_3_O_4_ surfaces in H_2_ atmosphere. Pt: dark blue; Mn: light violet; O: red; H: cyan. Source data are provided as a Source Data file.

Previous studies have shown that doping^29^, molecular carriers^11,30^, spectator molecules^25^, interface length^17^ and others can regulate the surface hydrogen migration amount, distance, and rate. However, the correlation between intrinsic properties of the oxide surface and the rate of hydrogen spillover has not been clearly understood. Figure 4a, b displays the normalized OD and O_L_ contents of stripe-like MnO and grid-like Mn_3_O_4_ as a function of D_2_ exposure amount. The normalized O_L_ contents of stripe-like MnO as a function of D_2_ exposure amount can be fitted using a *two parallel sites* model^31^: *θ* = 200.75 × 0.001 × e^−0.001 × *n* L^ + 11.1 × 0.07 × e^−0.07 × *n* L^ (n: D_2_ exposure amount; L = 10^−6^ mbar·s). This means that two kinds of hydrogen diffusion pathways exist on the stripe-like MnO/Pt(111) surface. According to the in situ STM experiment, the hydrogen diffusion along the stripe is the dominant pathway compared with that across the stripe. Hydrogen spillover rate as a function of hydroxylation degree can be derived from the slope of the profile of normalized lattice oxygen contents as a function of D_2_ exposure amount shown in Fig. 4a. When the exposure amount is <35 L (hydroxylation degree <70%), the hydrogen spillover rate remains nearly unchanged. With the H_2_ exposure amount is >35 L, the rate of hydrogen spillover decreases rapidly with the increasing degree of hydroxylation. The normalized O_L_ contents of grid-like Mn_3_O_4_ as a function of D_2_ exposure amount can be fitted using a *single site* model^31^: *θ* = 76.5 × 0.01 × e^−0.01×*n* L^ (n: D_2_ exposure amount; L = 10^−6^ mbar·s), implying that only one hydrogen diffusion pathway exists. As indicated by the slope of profiles in Fig. 4b, the rate of hydrogen spillover keeps on decreasing with the increasing degree of hydroxylation. In addition, on the D_2_ exposure traces, crossing points are observed at 16 L for MnO and 52 L for Mn_3_O_4_, indicating that hydrogen atoms diffuse faster on stripe-like MnO than on grid-like Mn_3_O_4_. We also studied the isotopic effect of spillover by using H_2_. As shown in Supplementary Fig. 4, the hydroxylation extent of the MnO and Mn_3_O_4_ films in H_2_ is slightly higher compared with the case in D_2_. This indicates the existence of a normal kinetic isotopic effect (*k*_*H*_/*k*_*D*_ > 1)^32,33^.

In order to investigate the relationship between hydrogen spillover rate and H_2_ partial pressure, in situ STM experiments were conducted. Firstly, stripe-like MnO overlayer was exposed to H_2_ at room temperature. By comparing the lengths of the bright lines at different exposure time, the growth rates can be calculated, which represents the hydrogen spillover rates. The initial hydrogen spillover rates were calculated under different H_2_ partial pressures, including 8.0 × 10^−9^, 2.3 × 10^−8^, 4.7 × 10^−8^, 1.6 × 10^−7^, and 3.0 × 10^−7^ mbar H_2_, and the logarithms of initial spillover rates vs. logarithms of ${p}_{{H}_{2}}$ are summarized in the orange line in Fig. 4c. The logarithms of hydrogen spillover rates show a half-order dependence on logarithms of H_2_ partial pressure (gradient 0.45) at room temperature, suggesting that the hydrogen diffusion is the rate-determining step.

The hydrogen spillover rates of grid-like Mn_3_O_4_ in different H_2_ partial pressures were investigated afterwards. By comparing the widths of the bright rim at different dosing times in the initial stage, the hydrogen spillover rates can be estimated. The initial hydrogen spillover rates were calculated in 9.3 × 10^−8^, 1.9 × 10^−7^, 5.3 × 10^−7^, 7.9 × 10^−7^, and 1.0 × 10^−6^ mbar H_2_ and the logarithms of initial spillover rates vs. logarithms of ${p}_{{H}_{2}}$ are shown in the dark cyan line in Fig. 4c. The fitted curve shares nearly the same gradient (0.47) as that of stripe-like MnO. Notably, the hydrogen diffusion rates on stripe-like MnO are four times faster than those on grid-like Mn_3_O_4_. This is probably accelerated by the 1D surface-lattice-confinement effect. The comparison of the spillover directions and rates on the two surfaces is illustrated in Fig. 4d. In addition, on both stripe-like MnO surface and grid-like Mn_3_O_4_ surface, hydrogen diffuses faster with the increasing H_2_ partial pressure, which highlights the demanding of in situ characterization of catalysts under atmospheres.

### Theoretical insights into hydrogen spillover

**Fig. 5:**
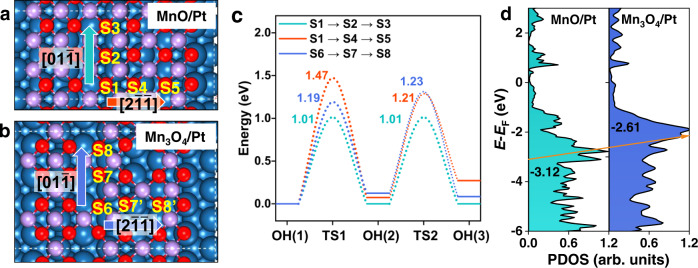
Theoretical analysis on difference of hydrogen diffusion over the MnO and Mn_3_O_4_ monolayers. Proposed monolayer (**a**) MnO and (**b**) Mn_3_O_4_ films supported on Pt(111) substrate. H: white; O: red; Mn: light violet; Pt: dark blue. Here, the characteristic surface O sites are denoted by yellow symbols and the hydrogen diffusion directions are indicated by colored arrows. **c** Potential energy diagram for O-H diffusion from site to site corresponding to (**a**) and (**b**). The zero energy level is relative to that of the first OH(1) state (i.e., H* on S1 and S6). The colored numbers denote the barriers for each elementary step. **d** Projected density of states (PDOS) of surface O sites (S1 and S6) in MnO/Pt(111) and Mn_3_O_4_/Pt(111), where the *p*-band centers of O are shown by the inserted numbers and their shifting direction is denoted by the orange arrow.

To gain insights into the difference of hydrogen diffusion on MnO and Mn_3_O_4_ surfaces, DFT calculations were carried out to obtain the energetics and electronic characters as shown in Fig. 5. According to our STM image data and the lattice mismatches^20,22^, two kinds of MnO_*x*_ ribbons on Pt(111), as shown in Figs. 1c, f, 5a, b, i.e., MnO/Pt(111) and Mn_3_O_4_/Pt(111), are utilized to simulate the local domains of the experimentally observed MnO and Mn_3_O_4_ monolayers.

For the monolayer MnO/Pt(111) surface (Fig. 5a), due to the reconstruction induced by the MnO-Pt(111) mismatch there are two distinct pathways for hydrogen diffusion from one to another O sites (termed S_n_, n is the designated number), including along the $[01\bar{1}]$ direction (S1 → S2 → S3) and the $[2\bar{11}]$ direction (S1 → S4 → S5). Figure 5c, Supplementary Fig. 5a and Supplementary Fig. 5b show that along the $[01\bar{1}]$ direction the barriers for hydrogen diffusion from S1 to S2 and from S2 to S3 are nearly the same, i.e., 1.01 eV, while along the $[2\bar{11}]$ direction hydrogen diffusion needs to overcome a barrier of 1.47 eV from S1 to S4 and 1.21 eV from S4 to S5. This indicates that hydrogen diffusion on MnO/Pt(111) is of a direction selectivity where the $[01\bar{1}]$ direction is preferential with a barrier of ~1.0 eV. We also consider a possibility of hydrogen diffusion via the Mn-H* intermediate but it is excluded due to its high barrier of 2.37 eV (Supplementary Fig. 6). Such results well explain the experimental observation that hydrogen unidirectionally diffuses on the MnO/Pt(111) surface (Fig. 2c–h), i.e., along the $[01\bar{1}]$ direction and through a OH-to-OH mode. As for the Mn_3_O_4_/Pt(111) surface (Fig. 5b, c and Supplementary Fig. 5c), along the $[01\bar{1}]$ direction the barrier for hydrogen diffusion across the Mn vacancy from S6 to S7 is 1.19 eV and that from S7 to S8 is 1.23 eV, both of which are higher than that of ~1.0 eV for the preferential pathway on MnO/Pt(111). This means that hydrogen diffusion on MnO/Pt(111) is much easier than that on Mn_3_O_4_/Pt(111), agreeing well with our XPS and STM results (Fig. 4).

We further aim to understand the nature regarding the difference of hydrogen spilling over MnO/Pt(111) *vs*. Mn_3_O_4_/Pt(111). Figure 5d shows the *p*-orbital projected density of states (PDOS) of various O sites on MnO/Pt(111) and Mn_3_O_4_/Pt(111), where a upshift of O *p*-band center (*ε*_p_) from −3.12 eV on MnO/Pt(111) to −2.61 eV on Mn_3_O_4_/Pt(111) is found. In some cases, the activity of O atom in oxides can be described by the *p*-band center, namely that the upshifting (more positive) of *ε*_p_ corresponds to a higher activity^34,35^. The movement of *ε*_p_ just corresponds to a change of coordination number from O_4c_ (4c denotes four-coordination) on MnO/Pt(111) to O_3c_ on Mn_3_O_4_/Pt(111), implying an increased activity of O. Through the binding energy calculation, we find that the adsorption energy of H on MnO/Pt(111) is −0.39 eV while that on Mn_3_O_4_/Pt(111) is −1.13 eV, indicating a higher stability and then a harder diffusion for H* on Mn_3_O_4_/Pt(111) than on MnO/Pt(111). Therefore, we suggest that hydrogen diffusion depends on the O-H* stability which intrinsically lies in difference of the activity of surface O atoms as indicated by the *p*-band center or the coordination number. In addition, we deduce that a medium O-O distance along the 1D direction (lattice confinement) is favorable for H diffusion, such as 2.77 Å (Fig. 1c, f), which implies a geometric effect beyond the coordination number. The (001) facets of single crystal (abbreviation as sc) MnO and Mn_3_O_4_ were constructed to investigate the influence of the interaction between Pt substrate and MnO_*x*_ monolayers toward the spillover rate. Comparing with the diffusion barriers of 1.23 eV on sc-MnO(001) and 1.57 eV on sc-Mn_3_O_4_(001) surfaces (Supplementary Fig. 7), we conclude that Pt substrate can promote H diffusion on the monolayer MnO_*x*_ films via the strong MnO_*x*_ and Pt interaction.

By extension, the effect of hydroxylation degree on H diffusion is further investigated. Supplementary Fig. 8 shows the H diffusion on the hydroxylated MnO_*x*_/Pt surface (the model with one H atom adsorbed on the adjacent O site). For MnO, the barrier for H diffusion is 1.06 eV, which is slightly higher than that on H-free surface (1.01 eV). For Mn_3_O_4_, H diffusion needs to surmount a 1.24 eV barrier near to those on H-free surface (1.19 or 1.23 eV). This implies that when H coverage is low the effect of hydroxylation on H diffusion is trivial in the two film systems. However, if hydroxylation induces the geometric evolution from the tetragonal (t-MnO/Pt) to hexagonal (h-MnO/Pt) phases (Supplementary Fig. 8b), H diffusion would be harder with a barrier of 1.78 eV. We thus infer that the reduced hydrogen spillover rate with the increasing hydroxylation degree as observed in experiments (Fig. 4a, b) may stem from the structural evolution induced by hydroxylation.

It is generally accepted that molecules confined in 1D spaces, such as carbon nanotubes^36^, zeolites^37^, metal organic frameworks^38^, and covalent organic frameworks^39^, diffuse along the 1D channels with enhanced transport properties. By adjusting the size of the confinement space and the strength of the host-guest interaction, diffusion behavior of molecules can be effectively modulated. However, confined diffusion of molecules on an open space or surface has been rarely reported. One example is that molecules diffuse on TiO_2_(110) surface preferentially along the shorter surface unit cell vector [001] direction^40–44^. As an open surface, stripe-like MnO(001) surface can regulate the hydrogen species to diffuse only along the shorter unit cell vector $[01\bar{1}]$ direction, which derives from 1D surface-lattice-confinement effect. More importantly, hydrogen spillover rates are accelerated by the 1D surface-lattice-confinement effect, which has not been reported previously. During this process, the geometric effect plays a dominant role in H diffusion which further supports that the 1D surface-lattice-confinement effect accelerates H diffusion on monolayer MnO.

## Discussion

In summary, stripe-like MnO(001) and grid-like Mn_3_O_4_(001) monolayers are constructed on Pt(111) substrate. In situ HP-STM and XPS investigations show that the surface-lattice-confinement effect can regulate the hydrogen spillover directions and accelerate the spillover rates. DFT calculations indicate that this is intrinsically related to the different local surface geometries and coordination numbers of surface O sites in the two systems. These findings illustrate the effect of surface structure on the kinetics of hydrogen spillover in oxide systems. It deepens our understanding of the factors that influence the rate of hydrogen spillover, which in some cases is the rate-determine step during the hydrogenation or dehydrogenation process on oxide catalysts.

## Methods

### Sample preparations

Pt(111) substrate (MaTeck) used for manganese oxides growth was cleaned by cycles of 1.3 keV Ar^+^ sputtering and annealing at 800 K in O_2_ atmosphere, followed by annealing at 1000 K in UHV. Manganese oxides were deposited by evaporating Mn shots in a Knudsen cell (Createc) in O_2_ atmosphere. All gases were purified with liquid N_2_ for more than 30 min before usage. The nominal MnO_*x*_ coverage is obtained from the statistical STM images.

### Characterizations

All characterizations were conducted in two UHV systems. One consists of a sample preparation chamber and HP-STM (SPECS, Germany) with a base pressure <3 × 10^−10^ mbar. HP-STM uses a mechanically cut Pt-Ir tip. For in situ STM experiments, the imaging experiments were conducted at room temperature. STM images were obtained in the constant current mode and processed by SPIP (Image Metrology, Denmark). The other is an Omicron multiprobe system, which is equipped with a sample preparation chamber (base pressure <5 × 10^–10^ mbar), a spectroscopic chamber (base pressure <3 × 10^–11^ mbar), and a microscopic chamber (base pressure <3 × 10^−10^ mbar). The spectroscopic chamber is equipped with XPS (Omicron, NG DAR 400), and core-level spectra are acquired using Al Kα (hν = 1486.6 eV) radiation and a hemispherical energy analyzer (Omicron, EA 125 U7). XPS measurements were conducted after the as-prepared MnO/Pt(111) and Mn_3_O_4_/Pt(111) surfaces were exposed to different amount of D_2_ at room temperature. The as-prepared MnO/Pt(111) surface was successively exposed to 1 × 10^−7^ mbar D_2_ for 50 s, 1 × 10^−7^ mbar D_2_ for 50 s, 1 × 10^−7^ mbar D_2_ for 50 s, 1 × 10^−7^ mbar D_2_ for 100 s, 1 × 10^−7^ mbar D_2_ for 100 s, 1 × 10^−7^ mbar D_2_ for 200 s, 5 × 10^−7^ mbar D_2_ for 80 s, 5 × 10^−7^ mbar D_2_ for 180 s, 5 × 10^−7^ mbar D_2_ for 360 s, and 1 × 10^−6^ mbar D_2_ for 300 s. The corresponding exposure amount of D_2_ was 5, 10, 15, 25, 35, 55, 95, 185, 365, and 665 L. XPS measurements on Mn_3_O_4_/Pt(111) were conducted in two experiments. The as-prepared Mn_3_O_4_/Pt(111) surface was successively exposed to 5 × 10^−7^ mbar D_2_ for 30, 30, and 30 s. Another as-prepared Mn_3_O_4_/Pt(111) surface was successively exposed to 5 × 10^−7^ mbar D_2_ for 120, 120, 120, 120, and 120 s. Accordingly, the exposure amount of D_2_ was 15, 30, 45, 60, 120, 180, 240, and 300 L. The normalized OD contents = $\frac{{{{{{{\rm{Area}}}}}}}_{{{{{{\rm{OD}}}}}}}}{{{{{{{\rm{Area}}}}}}}_{{{{{{\rm{OD}}}}}}}+{{{{{{\rm{Area}}}}}}}_{{{{{{{\rm{O}}}}}}}_{{{{{{\rm{L}}}}}}}}}$; the normalized O_L_ contents = $\frac{{{{{{{\rm{Area}}}}}}}_{{{{{{{\rm{O}}}}}}}_{{{{{{\rm{L}}}}}}}}}{{{{{{{\rm{Area}}}}}}}_{{{{{{\rm{OD}}}}}}}+{{{{{{\rm{Area}}}}}}}_{{{{{{{\rm{O}}}}}}}_{{{{{{\rm{L}}}}}}}}}$. All XPS spectra were analyzed by CasaXPS software with a Linear background subtraction and Gaussian-Lorentzian fitting. The binding energy of O 1*s* is corrected by using Pt 4*f* as a reference.

### Computational parameters

Spin-polarized DFT calculations were implemented using a plane-wave basis set in the Vienna Ab-initio Simulation Packages (VASP 5.4)^45^. The exchange-correlation energy was treated using Perdew-Burke-Ernzerhof (PBE) functional within the generalized gradient approximation (GGA)^46^. The projected-augmented wave (PAW) pseudopotentials were utilized to describe the core electrons, and a cutoff energy of 400 eV was used for the plane-wave expansion^47^. The van der Waals (vdW) dispersion forces were corrected by the vdW-DF (optPBE) function, which showed highly accurate description for oxides^48^. An on-site Hubbard term U_eff_ = U – J was added to address the open-shell *d*-electrons with 3.7 eV for Mn in the MnO_*x*_/Pt(111) system^49^. The water-based reference state for the calculations to avoid incorrect description of the gas phase O_2_ reference with standard DFT methods^48^. The energies and residual forces were converged to 10^−5^ eV and 0.02 eV•Å^−1^, respectively. The searching of transition states (TSs) is through the climbing image nudged elastic band (CI-NEB) method^50^.

### Model constructions

With consideration of the MnO/Pt(111) morphology and the lattice mismatch, a slab model of a (5√3 × 3) rectangular supercell was used, where a monolayer (6 × 3) MnO(001)-like ribbon was supported on three Pt(111) metal layers (20 Pt atoms in each layer) (Fig. 1c). This MnO/Pt(111) model is near to a “3” truncation of the “2-3-3” sequence, i.e., the (19 × 1) reconstruction observed by STM^20^. The optimized lattice constants are 2.82 Å for p(1 × 1) Pt(111), which is about 1.02 times than the experimental value (2.77 Å) due to the systematic error from DFT calculation, and 2.97 Å for free-standing p(1 × 1) MnO(001) monolayer, respectively. Thus, the MnO ribbon on Pt(111) suffers from an about −5.1% compressive strain along the $[01\bar{1}]$ direction relative to the free-standing one. As for the monolayer Mn_3_O_4_/Pt(111) surface which is still ill-defined by STM, we first obtained a series of reconstructed films (bridged Mn_2c_ transforms into quadruple Mn_4c_, 2c denotes two-coordination) by optimizing the free-standing monolayer Mn_3_O_4_(001) (Supplementary Fig. 9a) with different sizes, including the (1 × 1), (2 × 2), and (3 × 3) supercells (Supplementary Fig. 9b–d). We find that the reconstruction modes for Mn_2c_ are of difference among the three supercells, i.e., “A..B..C”, “A..A..B..B”, and “A..B..A” modes, respectively (Supplementary Fig. 9). We thus speculate that there may be more reconstruction modes and the experimental monolayer Mn_3_O_4_ should feature one of the Mn_2c_ reconstructions. This motivates us to propose an Mn_3_O_4_ ribbon consisting of six columns of Mn and O atoms on a (5√3 × 4) Pt(111) surface as shown in Fig. 1f, which is on the basis of the “A..B..A” reconstruction and whose Mn_3_O_4_ overlayer is of nearly 0.9% tensile strain along the $[01\bar{1}]$ direction. In addition, we utilize the (001) facets of single crystal MnO (Fm$\bar{3}$m, no. 225) and Mn_3_O_4_ (I4_1_/amd, no. 141) to mimic the extreme situation of MnO_x_ multilayers grown on Pt. For Brillouin zone integration, we employ (12 × 12 × 12) and (8 × 8 × 1) k-point grids within the Morkhorst-Pack scheme for Pt bulk and MnO(001) monolayer, respectively. For other films, facets, and hybrid MnO_*x*_/Pt(111) interfaces, equivalent k-point grids are utilized. The vacuum layer is set as over 13 Å to avoid the spurious interation between slabs. In addition, the magnetism for the MnO_*x*_/Pt(111) is simply set as the antiferromagnetic (AFM) ordering with consideration of its negligible influence on the tendency^51^.

Notably, we find the interface matching is clearly different between Pt(111) and MnO *vs*. Mn_3_O_4_ monolayers, where the monolayer MnO exhibits stronger adhesion to the Pt substrate through more Mn-Pt bonding (Fig. 1c). We thus assume that there may be an intrinsic difference for H diffusion along the $[2\bar{11}]$ and $[01\bar{1}]$ directions for the MnO/Pt(111) surface due to the reconstruction, whereas for Mn_3_O_4_/Pt(111) the two diffusion directions should be the same if excluding the edge effect limited by the ribbon model. We also note that such Mn_3_O_4_ monolayer is equivalent to the tetragonal MnO monolayer with a certain concentration of Mn defects, and there is an apparent difference on the coordination numbers of O, i.e., 3 for Mn_3_O_4_ and 4 for MnO, respectively.

### Formulae

1. The *p*-band center (*ε*_p_)^42^ of active O site is defined as
  1 $${\varepsilon }_{p}=\frac{{\int }_{-{{\infty }}}^{{{\infty }}}{n}_{p}\left(\varepsilon \right)\varepsilon d\varepsilon }{{\int }_{-{{\infty }}}^{{{\infty }}}{n}_{p}\left(\varepsilon \right)d\varepsilon }$$
2. The binding energy for H on the MnO_*x*_/Pt(111) (*E*_ads_) is calculated by

2 $${E}_{{ads}}(H)={E}_{H*}-{E}_{*}-{\frac{1}{2}}{E_{{H}_{2}}}$$

where ${E}_{H*}$ and $E_ \ast$ are the total energies of the H-adsorbed and the pristine surfaces, respectively.

## Supplementary information

Supplementary Information

Peer Review File

Description of Additional Supplementary Files

Supplementary Movie 1

Supplementary Movie 2

## Data Availability

Relevant data supporting the key findings of this study are available within the paper and the supplementary information file. Source data are provided with this paper. Source data are provided as a Source Data file. Source data are provided with this paper.

## Source data

Source Data
