# Supplementary material for: Direct observation of accelerating hydrogen spillover via surface-lattice-confinement effect
Source: Nat Commun. 2023 Feb 4;14:613. doi: 10.1038/s41467-023-36044-8 (PMC9899253; doi:10.1038/s41467-023-36044-8)
Supplement: Supplementary file 1 — Supplementary Information [file 41467_2023_36044_MOESM1_ESM.pdf]

Supplementary Information

Direct Observation of Accelerating Hydrogen Spillover via Surface-Lattice-Confinement Effect

Yijing Liu[1,2,†], *Rankun Zhang*[1,3,†], *Le Lin*[1,†] et al

Supplementary Figures

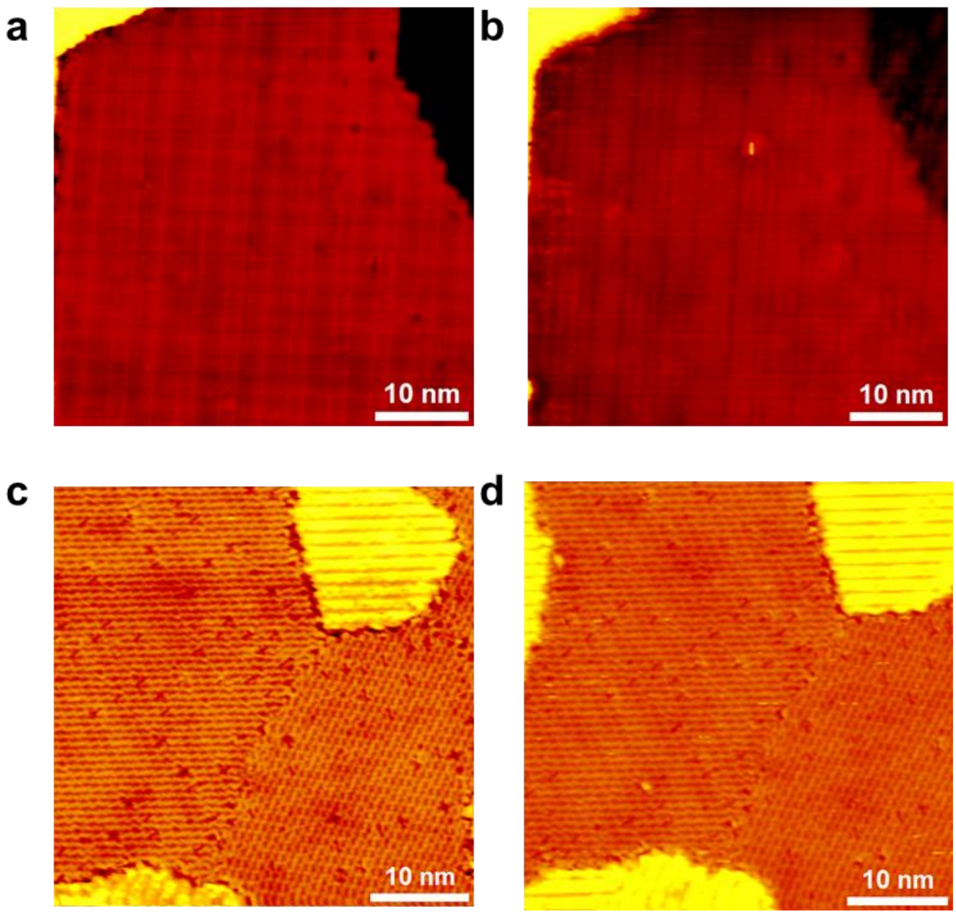

Supplementary Figure 1. STM images of 1.2 ML manganese oxide films before and after the exposure of H_2. (**a**) As-prepared 1.2 ML stripe-like MnO/Pt(111) and (**b**) the surface exposed to 1×10^{-6} mbar H_2, (**c**) as-prepared 1.2 ML grid-like Mn_3O_4/Pt(111) and (**d**) the surface exposed to 2×10^{-6} mbar H_2. Scanning parameters: (**a, b**) I_t = 0.090 nA, V_s = 0.940 V; (**c**) I_t = 0.090 nA, V_s = 0.806 V; (**d**) I_t = 0.070 nA, V_s = 0.806 V.

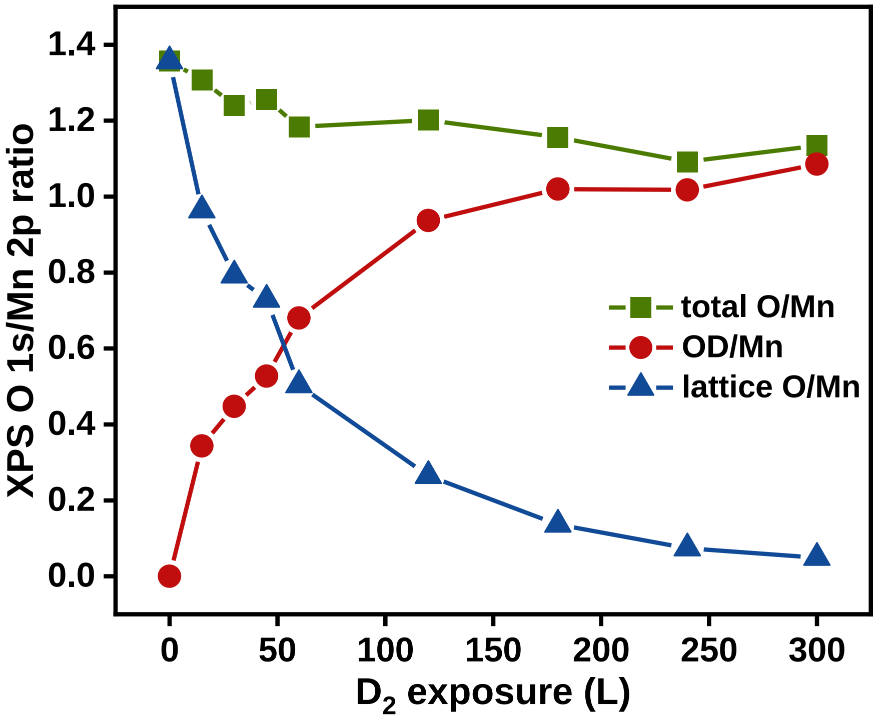

Supplementary Figure 2. XPS O 1s/Mn 2p peak area ratios of the grid-like Mn_3O_4 exposed to increasing amount of D_2 at room temperature. Source data are provided as a Source Data file.

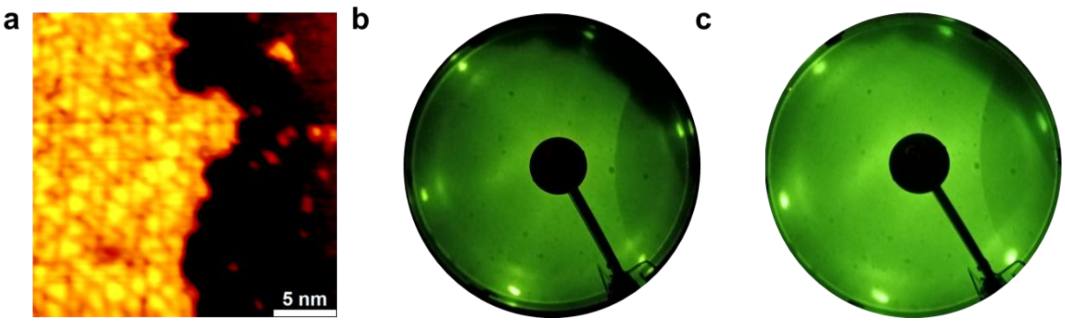

Supplementary Figure 3. Surface structure of hydroxylated MnO_x/Pt(111). (**a**) STM image of hydroxylated Mn_3O_4/Pt(111) surface. Scanning parameters: I_t = 0.100 nA, V_s = 0.969 V. (**b**) LEED patterns of hydroxylated stripe-like MnO/Pt(111), electron energy (E_{kin}) = 45 eV. (**c**) LEED patterns of hydroxylated grid-like Mn_3O_4/Pt(111), E_{kin} = 46 eV. The LEED patterns indicate that the hydroxylated MnO_x/Pt(111) surfaces are hexagonal symmetry with 3.3 Å lattice parameters.

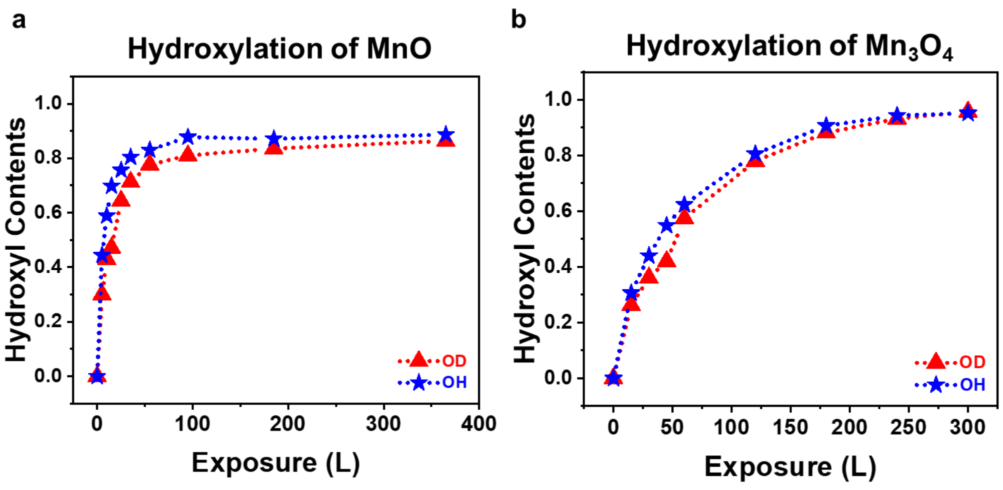

Supplementary Figure 4. Hydroxylation process of MnO and Mn_3O_4 films in D_2 and H_2 at room temperature. OD and OH contents derived from the XPS O 1s areas of (**a**) stripe-like MnO and (**b**) grid-like Mn_3O_4 surfaces with different D_2 and H_2 exposure. Source data are provided as a Source Data file.

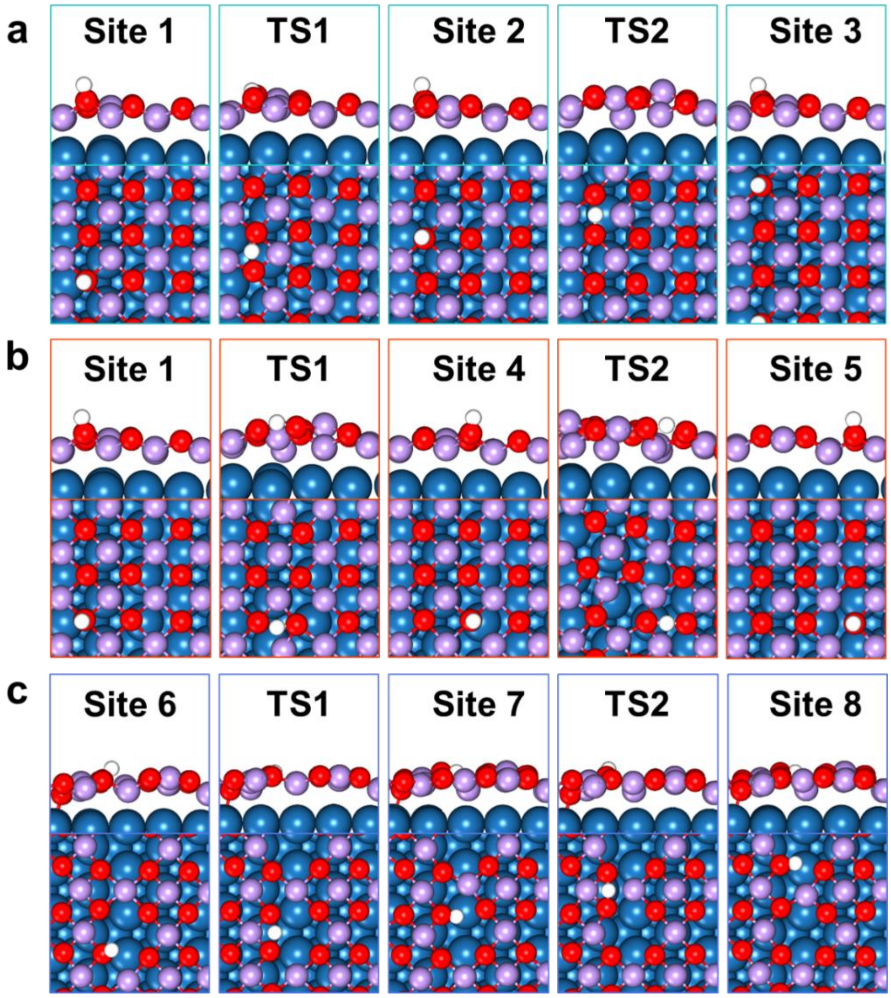

Supplementary Figure 5. Configurations (top and side views) of the H diffusion along different directions over the MnO/Pt(111) and Mn_3O_4/Pt(111) surfaces. (**a, b**) H diffusions along the two directions on MnO/Pt(111) as shown in Fig. 5a of the main text. (**c**) H diffusions along the $[01\bar{1}]$ direction on Mn_3O_4/Pt(111) as shown in Fig. 5b. H: white; O: red; Mn: light violet; Pt: dark blue.

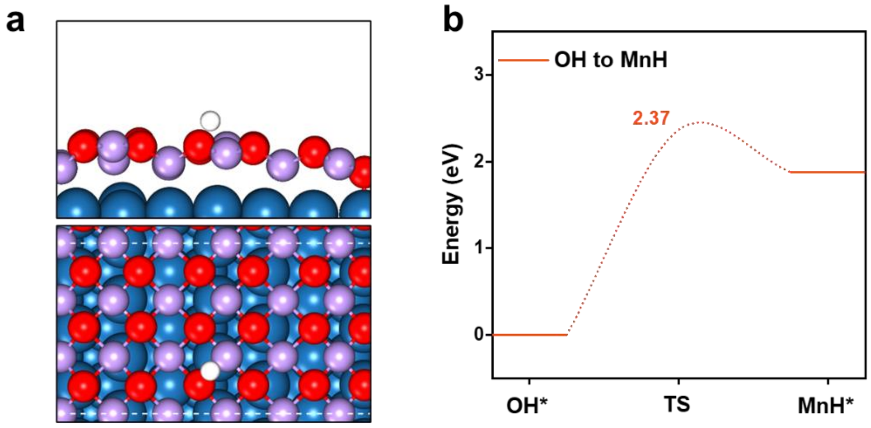

Supplementary Figure 6. Proposed H diffusion pathway from O-H* to Mn-H*. (**a**) Configuration (top and side views) of the transition state (TS) structure for O-H* to Mn-H* diffusion. H: white; O: red; Mn: light violet; Pt: dark blue. (**b**) Potential energy diagram for O-H* to Mn-H* diffusion. The inserted number denotes the barrier in eV.

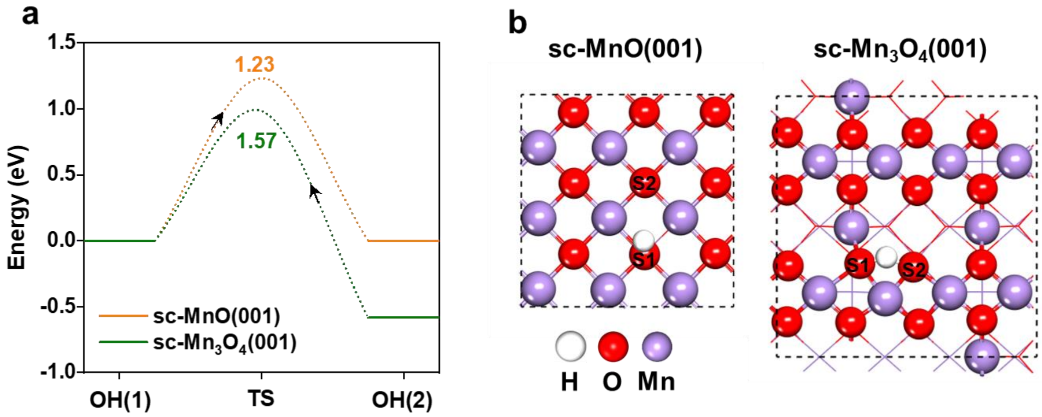

Supplementary Figure 7. H diffusion on the (001) facets of single crystal (abbreviation as sc) MnO and Mn_3O_4. (**a**) Potential energy diagram of H diffusion from one to another O sites. The barriers are calculated based on the H diffusion pathways along the arrow direction. (**b**) Configurations of the corresponding transition states. S1 and S2 represent two different O sites.

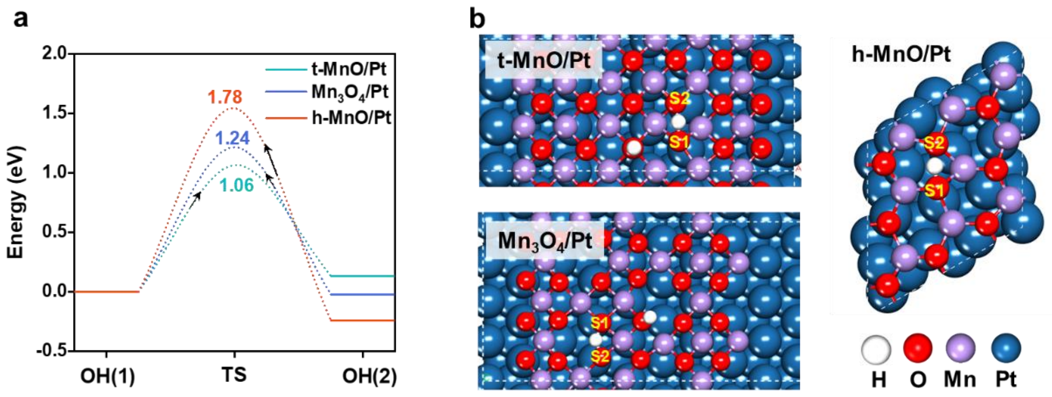

Supplementary Figure 8. H diffusion on the hydroxylated surfaces of tetragonal MnO/Pt (t-MnO/Pt) and Mn_3O_4/Pt, and the bare surface of hexagonal MnO/Pt (h-MnO/Pt). (**a**) Potential energy diagram of H diffusion from one to another O sites. The barriers are calculated based on the H diffusion pathways along the arrow direction. (**b**) Configurations of the corresponding transition states. S1 and S2 represent two different O sites.

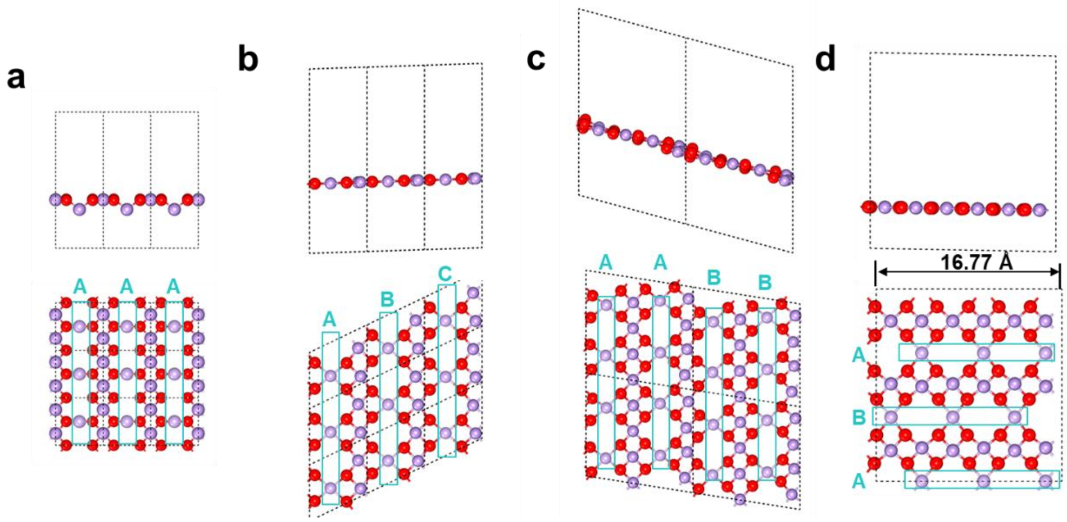

Supplementary Figure 9. Configurations of the H diffusion processes on MnO/Pt(111) and Mn_3O_4/Pt(111). (**a**) Pristine (3 × 3) Mn_3O_4 monolayer truncated from the spinel Mn_3O_4(001) surface. The dotted black line denotes the boundary of the unit cell. (**b-d**) Configurations (top and side views) of the

optimized Mn_3O_4 monolayers with various sizes, including the (1 × 1), (2 × 2), and (3 × 3) supercells. Here, the cyan rectangles highlight the reconstruction mode of the Mn_{2c} (2c denotes two coordinative) to Mn_{4c} as denoted by capital A. For example, the reconstruction mode for (**b**) can be recorded as "A..B..C". O: red; Mn: light violet.
