# Supplementary material for: Direct observation of accelerating hydrogen spillover via surface-lattice-confinement effect
Source: Nat Commun. 2023 Feb 4;14:613. doi: 10.1038/s41467-023-36044-8 (PMC9899253; doi:10.1038/s41467-023-36044-8)
Supplement: Supplementary file 3 — Description of Additional Supplementary Files [file 41467_2023_36044_MOESM3_ESM.pdf]

Description of Additional Supplementary Files

Supplementary Movie 1: 1D hydrogen spillover

Supplementary Movie 2: 2D hydrogen spillover
