# Supplementary figures and images for: Direct observation of accelerating hydrogen spillover via surface-lattice-confinement effect
Source: Nat Commun. 2023 Feb 4;14:613. doi: 10.1038/s41467-023-36044-8 (PMC9899253; doi:10.1038/s41467-023-36044-8)

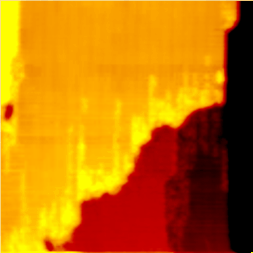

Supplement: Supplementary file 4 — Supplementary Movie 1 [file 41467_2023_36044_MOESM4_ESM.gif]

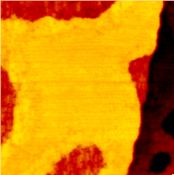

Supplement: Supplementary file 5 — Supplementary Movie 2 [file 41467_2023_36044_MOESM5_ESM.gif]
